# Guidelines for the conservative treatment of spinal deformities – Questionnaire for a Delphi consensus

**DOI:** 10.4102/sajp.v77i2.1587

**Published:** 2021-12-10

**Authors:** Elif E. Dereli, Shaopeng Gong, Tuğba Kuru Çolak, Deborah Turnbull

**Affiliations:** 1 Faculty of Health Sciences, Physiotherapy and Rehabilitation Program, Istanbul Bilgi University, Istanbul, Turkey; 2 Wuhan Schroth Scoliosis Service Center, Wuhan, Hubei, China; 3 Faculty of Health Sciences, Physiotherapy and Rehabilitation, Marmara University, Istanbul, Turkey; 4 School of Health and Social Care, University of Teesside, Middleborough, MA, United States of America; 5 The London Orthotic Consultancy, Kingston upon Thames, United Kingdom

**Keywords:** spinal deformities, conservative treatment, exercise, brace, Delphi consensus

## Abstract

**Background:**

Spinal deformity is the oldest disease known to humankind. Many types of treatment methods, including both conservative and surgical, are in use.

**Objective:**

We aimed to validate a published guideline protocol based on the conservative treatment of spinal deformities.

**Method:**

A modified Delphi technique was used with a questionnaire sent out to professionals worldwide regarding the conservative treatment of spinal deformities.

**Results:**

Our study was completed after two rounds. A strong level of agreement of 80% and more (consensus cut-off point) was achieved in most questions in the first round. Some statements were below this margin, and they were sent to the participants via email in the second round for re-evaluation. Consensus was achieved in almost all of the statements in the second round. Only two items did not reach the cut-off point but were close to this value.

**Conclusion:**

This proposed Guideline Protocol was approved by the participants using the Delphi method and can be used as a valid tool for the conservative treatment of spinal deformities.

**Clinical implications:**

A conservative treatment guideline in spinal deformity management, will provide consistency in treatment and will facilitate comparability with surgery. It will be useful in determining the cost-effectiveness of treatment and in choosing the right patient for the right method of treatment. This guideline might help in this context, and may also create a systematic method for clinicians to use as a reference in both research and clinical practice.

## Introduction

Spinal deformity is the oldest disease known to humankind (Kostuik 2015) The deformity in the adult spine may be ongoing for a long time or it may be a sequel to a developmental deformity experienced in childhood. There may also be a variety of contributing factors seen in childhood or adolescence. Thus, there may be many types of deformity involving the spinal vertebrae such as adult and adolescent forms of scoliosis, kyphosis, and lordosis (Diebo et al. 2019). Various treatment methods, including both conservative and surgical, have been applied to spinal deformities. Spinal alignment is important for maintaining an upright posture, protection of neural elements, and stability of the axial skeleton (Kim et al. 2020).

Scoliosis is defined as lateral deviation, axial rotation, and abnormal sagittal curvature of the spine, and is one of the most common spinal deformities. Hyper-kyphosis and lordosis are other types of sagittal plane spinal deformities often seen in clinical practice. All of these spinal conditions may lead to cosmetic appearance problems (Goldberg et al. 2001; Marks & Qaimkhani 2009; Weiss & Moramarco 2017), pain (Achar & Yamanaka 2020), postural imbalance (Herman et al. 1985), biomechanical deterioration (Schultz 1984) and respiratory dysfunction (Johari et al. 2016).

Many types of therapeutic approaches are being investigated but it is unclear which treatment is superior. Conservative treatment consists of exercise, which is broadly used (Negrini et al. 2003; Rigo, Reiter & Weiss 2003; Rivett et al. 2014; Weiss 2011; Weiss, Weiss & Petermann 2003), and bracing (Rivett et al. 2014; Weinstein et al. 2013). There is promising evidence regarding the efficacy of conservative treatment, especially exercise, in various spinal deformities (Anwer et al. 2015; Gür, Ayhan & Yakut 2017; Li et al. 2021; Liu et al. 2020; Park, Jeon & Park 2017; Parveen et al. 2020), and despite the growing high-quality evidence, the heterogeneity of the study protocols limits the generalisability of the recommendations (Negrini et al. 2018).

Surgery is another option; however, the long-term effects of surgery should be further considered. This option is much more invasive, with increased risk and in the long-term might be more problematic, given it does not always provide better results than conservative methods (Bettany-Saltikov et al. 2015; Romano et al. 2013). There is no evidence-based consensus on the superiority of either one of these options, namely conservative or surgery options (Acaroglu et al. 2017; Bettany-Saltikov et al. 2017; Kaspiris et al. 2011). Some evidence describes the importance and beneficial effects of different exercises (Berdishevsky et al. 2016; Negrini et al. 2015), but exercise needs a high level of patient compliance and should be performed as a disciplined routine.

Bracing is the most common conservative treatment option in spinal deformities. High quality studies exist supporting brace treatment during growth in patients with scoliosis (Weinstein et al. 2013; Weiss & Turnbull 2020b) and there is some evidence that standardised Chêneau style braces may be more sucessful than the more symmetric Boston style braces (Minsk et al. 2017; Weiss et al. 2020). Compliance with brace wearing, especially wearing the brace for sufficent time for potential benefit, is a key point (Rivett et al. 2014), because the benefit increases with longer hours of brace wearing (Weinstein et al. 2013). This may be stressful for patients who have to wear a brace for extended periods (Kuru & Yilmaz 2012). Brace support for pain and deformity might be indicated in adult patients with spinal deformities (Weiss & Turnbull 2019). Soft braces are not reported useful enough for stiff deformities (Weiss & Turnbull 2020b). There is evidence that pain can be successfully reduced with these bracing approaches, mainly affecting the sagittal profile (Weiss & Turnbull 2019). Adult patients with scoliosis can be tested explicitly for postural pain before the prescription of braces. In patients with larger deformities and those aiming to reduce their deformity, pattern-specific scoliosis braces are successful. It has been reported that there is no high-quality evidence to support brace treatment in adult patients with spinal deformities; however, the available evidence appears to be promising (Weiss & Turnbull 2020b).

Because there is a lack of validated guidelines outlining the importance and beneficial effects of conservative methods (Day et al. 2019), we decided to undertake our study to motivate clinicians to choose and understand conservative management. Guidelines are accepted as important tools in evidence-based practice that can reduce healthcare variation and improve patient outcomes (Woolf et al. 1999). Clinical practice guidelines are systematically developed statements that intend to assist clinicians and patients in making decisions about appropriate healthcare in specific circumstances (Field & Lohr 1992). To date, there is no gold standard for the treatment of spinal deformities, because there are not many studies comparing the effects of therapeutic interventions, namely surgery, bracing, and conservative methods such as exercise that includes specific exercises. It is important to have validated guidelines to create a systematic conservative treatment methodology.

There are no adequate and suitable comparable studies that help us to make a choice between exercise and surgical applications. An accurate assessment should be made considering the suitability of surgery for patients, and the risks, long-term effects, and complications that may arise. A conservative treatment guideline, which includes systematic exercise and brace therapy, may provide consistency in treatment methods and facilitate comparisons with surgery. It will be of value to be able to compare the cost-effectiveness of these treatment options and this may help clinicians to choose the right patient for the most suitable treatment. Our questionnaire aimed to validate a published guideline protocol (Weiss & Turnbull 2020a) based on the conservative treatment of these types of deformities.

## Method

To provide validated guidelines for therapists and patients, a questionnaire containing 46 questions was provided and emailed to professionals worldwide regarding the conservative treatment of spinal deformities. This questionnaire was intended to be evaluated and validated and is derived from the guideline protocol (see Appendix 1) published after a thorough evaluation by the Schroth Best Practice Experts (Weiss & Turnbull 2020a).

We aimed to test the guideline protocol by using a modified Delphi method. The Delphi method was developed in the 1950s by two researchers, Olaf Helmer and Norman Dalkey, working at RAND (an organization formed immediately after World War II to connect military planning with research and≈development decisions) (RAND Corporation, 2021) in the USA, specifically to provide predictions on military issues. This method is widely used and accepted for achieving convergence of opinion concerning real-world knowledge solicited from experts within specific topic areas (Dalkey & Helmer 1963). It is a method that systematically obtains expert opinions on the subject in the presence of a problem. Thus, using the≈Delphi method can enable individuals and groups who look at a problem or clinical situation from different angles to reconcile their opinions without coming face to face. The use of the Delphi method often includes questionnaires applied sequentially to experts on a particular topic. After each round, the results are communicated to the participants. The process continues in this manner until consensus is reached. The consensus that is then achieved is the end product of this process (Dalkey & Helmer 1963; Patton 2008).

The questionnaire based on the conservative treatment of spinal deformities, which was derived from a protocol, contained three parts, including 21 general statements, 15 statements regarding indications for scoliosis, and 10 statements regarding indications for kyphosis treatment. The participants were informed that they could decide whether they agreed with each statement or not, using a level of agreement or disagreement (measuring scale from 1 (little agreement or disagreement) to 7 (strongest agreement or disagreement). There was consensus for this Modified Delphi method, which was revealed by the fact that 80% of respondents indicated that they ‘Strongly Agree’ with the statement, which means ‘I support this recommendation very strongly’. Thus, we accepted 80% and this included levels of agreement 5, 6, and 7 as the cut-off point, which means that these statements reached consensus.

The participants included medical doctors, physiotherapists, orthotists, personal trainers and others with a special interest in spinal deformities. They were the ones working with patients with spinal deformity who had both theoretical and practical knowledge and experience in the field. All participants had at least 2 years or more experience in this field. One hundred and forty-five international participants were invited to complete the questionnaire.The number of participants in Delphi studies varies, as there is no agreement as to how many people should ideally be included in a Delphi study (Murphy et al. 1998).

Contacted individuals received an email, including written explanations about the completion of the questionnaire, two attached documents (the questionnaire was sent as a word document and as a portable document (PDF) format) and a link for online questionnaires with a 2-week deadline for completion and return. In theory, the Delphi method can be iterated continuously until consensus is reached. However, it is noted that two or three rounds are usually sufficient to gather the necessary information and, in most cases, to reach consensus (Brooks 1979; Custer, Scarcella & Stewart 1999; Fernández-Llamazares et al. 2013).

We implemented a two-round modified Delphi method. The participants were informed that the data derived from the answered questionnaires was anonymous. Quality assurance was ensured by two academic physiotherapists who reviewed the data.

### Data analysis

Data analysis was performed after each Delphi round to provide statistical feedback to the participants and to determine whether consensus was reached. Data from each statement was collected using Microsoft Excel (Microsoft Corporation, Redmond, WA) and Statistical Package for the Social Sciences (SPSS) statistical software version 16.0 (SPSS Inc., Chicago, IL, USA) was used for the data analysis. Descriptive statistics, including means, standard deviations, medians, and percentages, were analysed for each statement.

Various methods and cut-off points have been determined to achieve consensus. In some studies, 51% of consensus is considered sufficient (Loughlin & Moore 1979); in some, 70% is accepted (Meshkat et al. 2014; Williams & Webb 1994) and in others 80% is accepted (Stewart et al. 2017; Veraar, Hasler & Schirmer 2018). The most common definition for consensus is 75% (Diamond et al. 2014). In our study, the consensus was defined as 80% or more of participants agreeing with certainty (at levels 5, 6, and 7).

## Results

**FIGURE 1:**
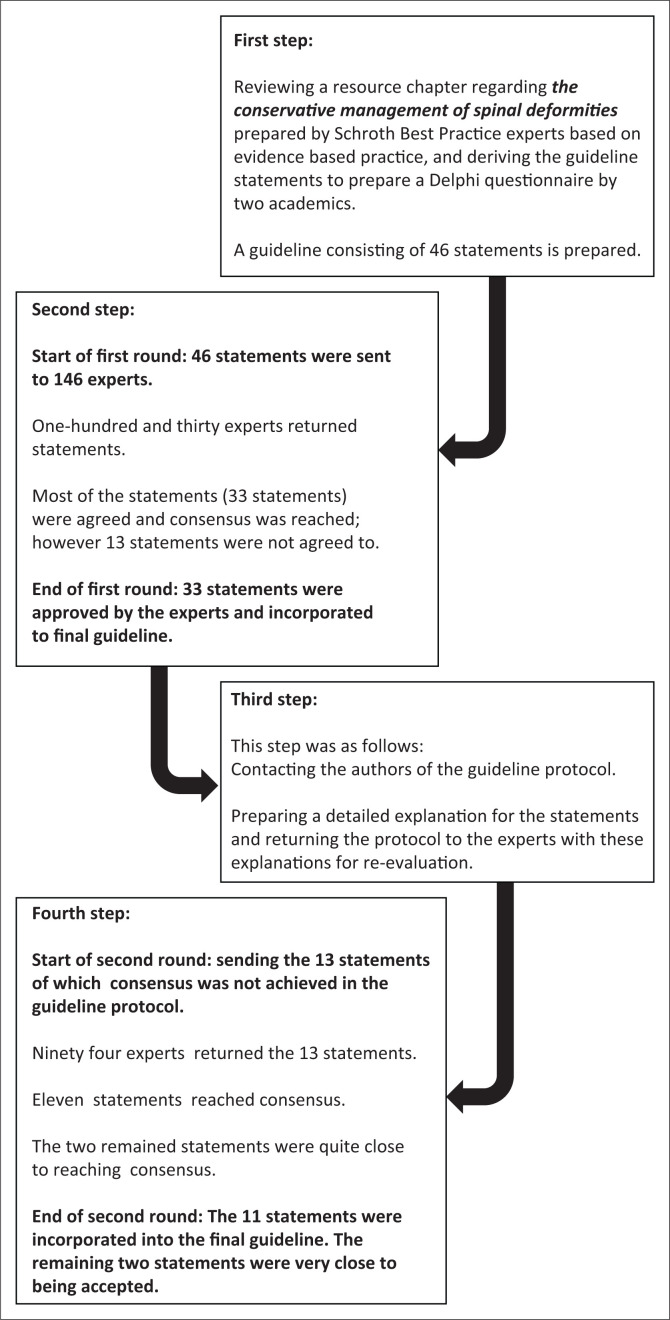
Flow diagram of our study.

In the first round, 130 participants (from 14 different countries, namely Canada, China, Cuba, Denmark, France, Germany, Greece, Indonesia, Japan, Thailand, Turkey, the United Kingdom, Ukraine, Australia) returned the completed questionnaires. The study process including the number of participants in each round and the status of the statements is shown in the Flow Diagram (Figure 1). The professions of the participants are given in Table 1, and the mean years of experience was 7.35 ± 7.83 years.

**TABLE 1:** Professions of the participants (*n* = 130).

Profession of the participants	Frequency	%
Physiotherapists	64	49.2
Medical doctors	30	23.1
Orthotists	18	13.8
Sports therapists	10	7.8
Chiropractors	6	4.6
Nurses	1	0.8
Osteopaths	1	0.8

**Total**	**130**	**100.0**

Most participants (67%) reported that they regularly treat all types of spinal deformities, including adult scoliosis, adolescent scoliosis, juvenile scoliosis, kyphosis, lordosis; and the rest of the participants reported that they regularly had contact with patients with at least one or more of these types of deformities. Ninety seven (75%) participants mentioned Schroth Therapy, 9 (7%) participants mentioned Schroth Best Practice, as therapy methods; 51 (39%) mentioned bracing and 41 (32%) mentioned the use of chiropractic techniques. Many other types of treatment models such as dry cupping, massage, sports rehabilitation, swimming, ‘Spiral Stabilization of the Spine (SPS)’, and acupuncture were reported by a small number of participants.

### First round

**FIGURE 2:**
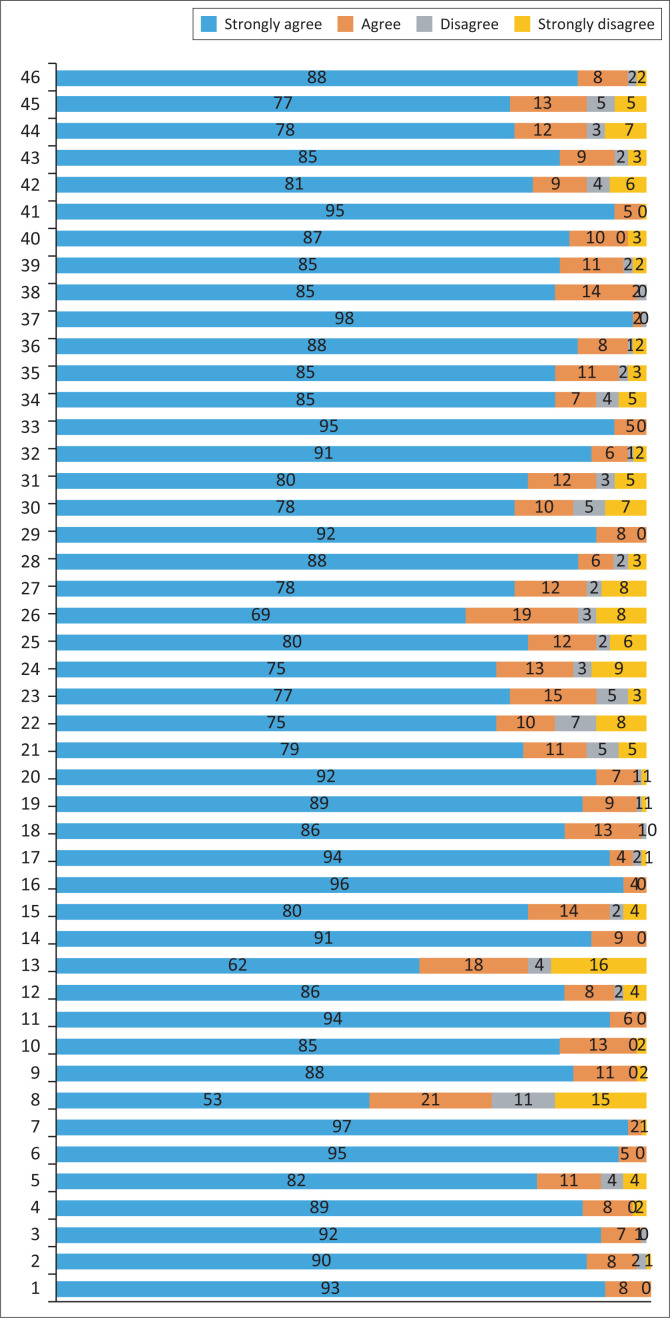
Levels of agreement and disagreement for each statements after first round.

The response rate for the first round was 130/145, that is 90% participation. A strong level of agreement 80% and above was achieved in most statements (33 out of 46) in the first round (Figure 2). However, some statements were below this margin, and these statements (Table 2) were prepared to be sent again via email to the participants for the re-evaluation process (Round 2).

**TABLE 2:** The statements of the guideline, mean, median values with standart deviations, and frequency of strong agreement levels and consensus situation in the first round.

The guideline statements	*X*	Standard deviation	Median	Frequency % of (5–7) very strong agreement	Consensus
The primary goal of scoliosis and kyphosis management in growing children of Risser 0 to Risser 3 is to stop curve progression and to try to improve curvature through growth.	6.39	1.22	7	92.3	Consensus was reached.
The primary goal of scoliosis and kyphosis management in older adolescents with less growth should be to improve cosmetic appearance and postural balance, whilst halting any further curve progression.	5.94	2.06	7	89.3	Consensus was reached.
Improving pulmonary function (vital capacity) and treating pain is also of major importance.	6.33	1.45	7	92.4	Consensus was reached.
Conservative scoliosis management is based on rehabilitative treatment and bracing.	6.02	2.20	7	89.2	Consensus was reached.
Today there is evidence for the effectiveness of scoliosis treatment using physical rehabilitation alone.	5.03	3.13	6	81.5	Consensus was reached.
Therapy for scoliosis does not just consist of general exercises.	6.53	1.03	7	95.4	Consensus was reached.
Methods specific to scoliosis requires that clinicians be specifically trained and certified in these targeted conservative intervention methods.	6.52	1.52	7	97	Consensus was reached.
Out-patient rehabilitation produces similar results to inpatient rehabilitation results and is effective at improving the common signs and symptoms of scoliosis and impeding curve progression.	2.76	4.62	5	**53.1**	**Consensus was not reached.**
Bracing is effective in preventing progression and improving curvature and in altering the natural history of idiopathic scoliosis.	5.95	1.88	7	87.7	Consensus was reached.
Brace treatment may reduce the prevalence of surgery, restore the sagittal profile, and influence vertebral rotation.	5.95	2.02	7	85.3	Consensus was reached.
Patient compliance is important for end-results of brace treatment.	6.49	1.09	7	93.8	Consensus was reached.
Rigid braces have superior end-results than soft braces.	5.79	2.87	7	86.2	Consensus was reached.
Simple deflection exercises can be performed in general to achieve a wider range of motion in kyphosis treatment.	3.39	4.76	5	**61.5**	**Consensus was not reached.**
Exercises and activities of daily living (ADLs) for patients with lumbar and thoracolumbar kyphosis are effective.	6.13	1.18	7	80.8	Consensus was reached.
Bracing is effective in preventing curvature progression and thus in altering the natural history of kyphosis.	5.21	2.94	6	80	Consensus was reached.
Each patient with scoliosis has their own natural history and must be considered on an individual basis in the context of a thorough objective clinical evaluation, patient subjective and on their past medical history.	6.60	0.93	7	96.1	Consensus was reached.
The risk of scoliosis progression highly correlates with the potential for growth.	6.29	1.88	7	93.9	Consensus was reached.
The progression factor should be calculated using the Lonstein and Carlson’s progression estimation formula in patients with high growth velocity.	6.09	1.44	7	86.2	Consensus was reached.
The treatment programme should be decided by calculating the progression risk according to the age and Cobb angle in patients with lower growth velocity.	6.10	1.75	7	89.2	Consensus was reached.
The indication for physical rehabilitation during the main growth spurt depends upon the individual and certain variables such as Cobb angle, apical curve location and Risser sign regarding the predicted treatment outcome in adolescent idiopathic scoliosis (AIS).	6.27	1.65	7	91.6	Consensus was reached.
Brace treatment is indicated and paramount to conservative management during growth and following the main growth spurt, physical rehabilitation can be effective independently of brace treatment in AIS.	4.92	3.52	6	79.2	**Consensus was not reached.**
Cobb angle up to 15° observation (6–12-month intervals).	4.27	4.13	6	74.7	**Consensus was not reached.**
Cobb angle 15° – 20°: Physical rehabilitation with treatment-free intervals (6–12 weeks without physical rehabilitation for those patients having low risk for curve progression at the time).	5.01	3.21	6	76.9	**Consensus was not reached.**
Cobb angle 20° – 25°: Physical rehabilitation.	4.53	4.00	6	74.6	**Consensus was not reached.**
Cobb angle > 25°: Physical rehabilitation and brace wearing part-time	5.10	3.49	6	79.9	**Consensus was not reached.**
Progression risk < 40%: Observation (3-month intervals).	4.38	3.88	6	**69.2**	**Consensus was not reached.**
Progression risk 40% – 60%: Physical rehabilitation.	4.85	3.65	6	77.7	**Consensus was not reached.**
Progression risk 60% – 80%: Physical rehabilitation + part-time brace indication (16 h – 23 h [low risk]).	5.64	2.74	7	88.4	Consensus was reached.
Progression risk > 80%: Physical rehabilitation + full-time brace indication (22 h full time – to reduce Cobb angle and improve cosmetic appearance through growth or 16 h – 18 h part time to halt curve).	6.30	1.30	7	91.5	Consensus was reached.
Cobb angle up to 20°: Observation (6–12 monthly intervals).	4.63	3.75	6	77.8	**Consensus was not reached.**
Cobb angle 20° – 35°: Physical rehabilitation.	5.15	3.36	6	79.9	**Consensus was not reached.**
Cobb angle > 35°: Physical rehabilitation + brace (22 h full time with aim to improve cosmetic appearance or 16 h – 18 h part time to halt curve – expectation is that the part-time wearer is not likely to improve their cobb angle at these later stages of growth).	5.92	2.21	7	90.8	Consensus was reached.
For brace weaning: Physical rehabilitation + brace with reduced wearing time.	6.37	1.09	7	94.6	Consensus was reached.
Cobb angle 25° – 35°: Physical rehabilitation.	5.20	3.28	6	84.7	Consensus was reached.
Cobb angle > 35°: Physical rehabilitation + brace (22 h full time if wanting to improve cosmetic appearance and or 16 h – 18 h part time to halt curve – expectation is that the part-time wearer is not likely to improve their cobb angle at these later stages of growth).	5.55	2.67	6,5	84.6	Consensus was reached.
Physical rehabilitation should be recommended.	5.89	2.37	7	88.5	Consensus was reached.
Treatment programme should include Physical rehabilitation, scoliosis rehabilitation programme (multimodal pain concept/behavioural + physical concept) and brace treatment.	6.72	1.10	7	97.7	Consensus was reached.
Brace treatment, like in other spinal deformities, is indicated when the curvature exceeds a Cobb angle of 40° in the thoracic area and when lumbar or thoracolumbar lordosis has vanished, and/or a kyphosis is visible in these areas.	5.75	1.66	6	84.6	Consensus was reached.
If there is inhibition of extension thoracic, thoracolumbar or lumbar: Physical rehabilitation.	5.66	2.51	6	85.4	Consensus was reached.
Cobb angle > 40° thoracic, any kind of thoracolumbar or lumbar kyphosis: Physical rehabilitation + brace (Minimum brace wear of 16 h per day).	5.76	2.45	7	86.9	Consensus was reached.
When weaning from brace: Physical rehabilitation + brace with reduced wearing time.	6.25	1.44	7	94.6	Consensus was reached.
Cobb angle is 40° – 50° thoracic, any kind of thoracolumbar or lumbar kyphosis: Physical rehabilitation.	4.92	3.52	6	80.8	Consensus was reached.
Cobb angle > 50° thoracic, > 10° of kyphosis thoracolumbar or lumbar: Physical rehabilitation + brace (16 h –18 h part time if wanting to improve cosmetic appearance and halt curve – expectation is that the part-time wearer is not likely to improve their curvature at these later stages of growth).	5.55	2.76	6,5	85.3	Consensus was reached.
Cobb angle > 50° thoracic, > 10° of kyphosis thoracolumbar or lumbar: Physical rehabilitation.	4.86	3.67	6	77.7	**Consensus was not reached.**
Physical rehabilitation, inpatient rehabilitation.	4.98	3.40	6	76.9	**Consensus was not reached.**
Physical rehabilitation, scoliosis rehabilitation programme (multimodal pain concept/behavioural + physical concept), brace treatment when a positive effect has been proven during specific testing.	5.86	2.26	7	88.4	Consensus was reached.

Note: Specific statements regarding scoliosis and kyphosis.
For Scoliosis:
In children (no signs of maturity, age 6–10 years): 22–25 In children and adolescents, Risser 0–3, first signs of maturation, less than 98% of mature height (bone age < 14 years – girls, < 16 years – boys) Type of Treatment provision: 26–29 In children and adolescents presenting with Risser 4 (more than 98% of mature height): 30–33 First presentation with Risser 4–5 (more than 99.5% of mature height before growth is completed): 34–35 Adults with Cobb angles > 30°: 36 Adolescents and adults with scoliosis (of any degree) and chronic pain: 37 (2) For Kyphosis: 38
Children and adolescents, Risser 0–3, first signs of maturation, less than 98% of mature height: 39–41 Children and adolescents presenting with Risser 4 (more than 98% of mature height):42–43 First presentation with Risser 4–5 (more than 99.5% of mature height before growth is completed):44 Adults with Cobb angles thoracic > 50°, > 10° of kyphosis thoracolumbar or lumbar:45 Adolescents and adults with kyphosis (of any degree) and chronic pain: 46

The results obtained in the first round and the questionnaire, were given to the participants for the second round of the Delphi process. They were asked to review their decisions in the first Delphi round and state any new decisions. Items that could not reach consensus within this period before the second round were re-examined. The Delphi method allows for participants to change their thoughts through written feedback. For this reason, details were requested from the authors who developed the proposed guideline for the items that were not agreed upon. Thus, more detailed explanations about the items were prepared by discussing them with the authors of the guideline from which the questionnaire was derived. Accordingly, the explanatory information (obtained from Schroth Best Practice Experts) for each statement that had not reached consensus was as follows (all of the statements are included in Table 1):

- Statement 8: The latest studies (randomised controlled studies) on exercise have been performed on an out-patient basis. Actually, there is no study on inpatient rehabilitation with the same or even better results.
- Statement 13: In daily practice, we regularly experience that a kyphosis can be easily mobilised with simple de-flexion / extension exercises.
- Statement 21: As currently no study exists using exercises the treatment option that demonstrates arresting curve progression in immature patients with Risser 0–2, Age 10 – 14, with Cobb angles of 25° – 40° followed until the end of growth. So exercises can be considered after the main growth spurt is over and treatment can primarily focus on bracing as long as the patients are at high growth velocity.
- Statements 22 – 25: These are patients with little growth where we usually try to avoid unnecessary effort at this stage in order to keep their motivation for the growth spurt.
- Statements 26, 27: With a calculated risk of < 40% I have good experience with only observation for 3 months periods and then adding physiotherapy with a calculated risk between 40% and 60% as well. We should keep in mind not to overload our patients, when it is not necessary, because we may need them to be motivated when their conditions are progressive.
- Statements 30, 31: Here we have little growth left which means little risk for progression and little chance to improve.
- Statements 44 and 45: Thoracic kyphoses in principle are more benign than scolioses. In the more mature patients as well as in adults physiotherpy approaches are sufficient and in my experience can contribute to improvement.

### Second round

In the second round , feedback was obtained from 94 participants, and the response rate was 94/130 which was 72% of the first round participants. Although there were losses compared to the first round, the number of participants returning for the second round was still enough to manage the Delphi method.

As in the first round, 80% consensus was achieved at the agreement values of 5,6, and 7. Only items that did not reach consensus in the first round were considered, and the results of the second round are presented in Table 3. It is clear from the second round values shown in Table 3, that consensus was achieved in all; but two of the statements for which consensus was not reached in the 1st round. These two items (items 8 and 22) did not reach the specified cut-off value but were close to it.

**TABLE 3:** The statements of the guideline, mean, median values with standard deviations, and frequency of strong agreement levels and consensus situation in the second round.

The guideline statements that did not reach a consensus in the 1st round	X	Standard deviation	Median	Frequency % of (5–7) very strong agreement	Second round consensus Situation
8. Out-patient rehabilitation produces similar results to inpatient rehabilitation results and are effective at improving the common signs and symptoms of scoliosis and impeding curve progression.	4.42	4.15	6	79.7*	Consensus was not reached. However this is a very close value, thus accepted.
13. Simple deflection exercises can be performed in general to achieve a wider range of motion in kyphosis treatment.	4.89	3.61	6	84	Consensus was reached.
21. Brace treatment is indicated and paramount to conservative management during growth and following the main growth spurt, physical rehabilitation can be effective independently of brace treatment in adolescent idiopathic scoliosis (AIS).	5.20	3.74	7	85.1	Consensus was reached.
22. Cobb angle up to 15° observation (6–12-month intervals).	4.38	4.51	6	78.8*	Consensus was not reached. However this is a very close value, thus accepted.
23. Cobb angle 15° – 20°: Physical rehabilitation with treatment-free intervals (6–12 weeks without physical rehabilitation for those patients having low risk for curve progression at the time).	5.44	2.98	6	87.3	Consensus was reached.
24. Cobb angle 20° – 25°: Physical rehabilitation.	5.03	3.95	7	84	Consensus was reached.
25. Cobb angle > 25°: Physical rehabilitation and brace wearing part-time	5.78	2.71	7	89.3	Consensus was reached.
26. Progression risk <40%: Observation (3-month intervals).	4.73	3.83	6	82.9	Consensus was reached.
27. Progression risk 40% – 60%: Physical rehabilitation.	4.92	3.75	6	80.9	Consensus was reached.
30. Cobb angle up to 20°: Observation (6–12 monthly intervals).	4.66	4.02	6	81.8	Consensus was reached.
31. Cobb angle 20° – 35°: Physical rehabilitation.	5.67	2.67	6	87.3	Consensus was reached.
44. Cobb angle > 50° thoracic, > 10° of kyphosis thoracolumbar or lumbar: Physical rehabilitation.	4.90	3.92	6	84	Consensus was reached.
45. Physical rehabilitation, inpatient rehabilitation.	5.28	3.24	6	86.1	Consensus was reached.

*Source:* Weiss, H.R. & Turnbull, D., 2020a, ‘Best practice recommendations for the conservative treatment of patients with spinal deformities’, in M. Borysov, M. Moramarco, S.Y. Ng & Weiss, H.R. (eds.), *Schroth’s textbook of scoliosis and other spinal deformities*, pp. 760–775, Cambridge Scholars Publishing, Newcastle upon Tyne

Note: Specific statements regarding scoliosis and kyphosis.
For Scoliosis
In children (no signs of maturity, age 6–10 years): 22–25 In children and adolescents, Risser 0–3, first signs of maturation, less than 98% of mature height (bone age < 14 years – girls, < 16 years – boys) Type of Treatment provision: 26–29 In children and adolescents presenting with Risser 4 (more than 98% of mature height): 30–33 First presentation with Risser 4–5 (more than 99.5% of mature height before growth is completed): 34–35 Adults with Cobb angles > 30°: 36 Adolescents and adults with scoliosis (of any degree) and chronic pain: 37 For Kyphosis:38
Children and adolescents, Risser 0–3, first signs of maturation, less than 98% of mature height: 39–41 Children and adolescents presenting with Risser 4 (more than 98% of mature height): 42–43 First presentation with Risser 4–5 (more than 99.5% of mature height before growth is completed): 44 Adults with Cobb angles thoracic > 50°, > 10° of kyphosis thoracolumbar or lumbar:45 Adolescents and adults with kyphosis (of any degree) and chronic pain:46 These statements around 79% agreement were so close to the 80% cut-off that they were also deemed to have reached consensus.

## Discussion

A well-structured and easy to access conservative therapy protocol may be of great help for both patients and professionals. These protocols have been established to help find a pathway to the most appropriate way of managing individual cases. Thus patients can monitor prescriptions and proposals made by professionals and test whether these are appropriate. Within this context, overtreatment and undertreatment might be avoided for both patients and professionals (Weiss 2010). The main goal of our study was to provide the most appropriate, feasible, effective, and safe management that does not impact the lives of these patients by taking up their time and their health resources.

Currently, promising evidence shows that it might be possible to be successful in treating patients conservatively without surgery by taking into account the nature and type of the spinal deformity and the eligibility of the patient. This guideline highlights the importance of non-operative management for eligible patients, given that surgery is an invasive procedure that may be associated with more risks than the conservative options (Acaroglu et al. 2017). To date there is no specific outcomes to explain patient satisfaction post surgery (Menendez et al. 2019), and thus it may not be appropriate to be presented as the first and best option for patients.

There is no evidence that suggests that conservative management of scoliosis will have a worse outcome than surgery, (Weiss et al. 2016; Weiss & Moramarco 2013). Patients should first be treated with a conservative programme and not referred to a surgeon initially. Prescription and adherence to an early rehabilitation programme has the potential to result in successful treatment. Surgery should be considered only after reaching a situation where the symptoms cannot be managed conservatively, as it is associated with a number of long-term complications (Bettany-Saltikov et al. 2017).

Establishing proven guidelines for treatment will contribute to a more systematic approach in the conservative treatment of spinal deformities. The systematisation of conservative treatments applied in this way will serve to create a strong alternative to surgery that may have both short- and long-term risks. The guidelines may lead to further studies that will provide better levels of evidence.

There is no evidence that includes a detailed and systematic approach to the conservative treatment of spinal deformities, but related recommendations can be seen in The International Society on Scoliosis Orthopaedic and Rehabilitation Treatment (SOSORT) consensus articles (De Mauroy, et al. 2010; Negrini et al. 2018; Weiss et al. 2006). This Delphi method has enabled some consensus on the conservative treatment of spinal conditions.

Consensus was achieved for all of the 46 statements of the guideline during the two Delphi rounds. The first round resulted in a consensus, with the majority (33 of 46 statements, 72%) of the statements being accepted. For the statements that did not reach agreement (13 of 46 statements, 28%), more detailed explanations had to be added to minimise misunderstanding or misinterpretation and to clarify the statements. Evaluation and feedback were provided by the authors about the statements for which no consensus was reached, and they were forwarded to the participants again with the authors’ permission. In the second round, the majority (11 of 13 statements, 85%) of the remaining statements reached consensus. The remaining two statements (statements 8 and 22), which did not reach consensus (but were close to the cut-off point) can possibly be considered to have reached consensus because of their high acceptance levels (Stewart et al. 2017). Thus, it may be considered that all three parts of the guideline were accepted.

Based on the statements suggested by the guideline and the opinions of its authors the following needs to be considered. In scoliosis, at stages of low growth dynamics (patients between 6 and 10 years, before the onset of the first signs of maturation), the risk of the patient becoming progressive is low; however, at the same time, there are low expectations for a recovery. So at this stage, therapy intensity can be reduced and only observation of patients with smaller curves needs to be done. Before these guidelines were drawn up, patients with curves of 20° – 35° were treated with full-time brace treatment before the main growth spurt, and often no improvement was seen. Patients then treated in this way are not sufficiently motivated at the decisive stage of puberty growth and tend not to wear braces at a time when this is most important. As a result, their curves are greatly distorted. Therefore, it may be considered that it is better not to stress patients during the stages of low growth dynamics, thereby mentally preparing them for the main growth spurt, and thus maintaining motivation for optimal treatment with full brace-wearing when appropriate. This is also the time where a clinician has the best chance for improvements, provided patients are treated with effective high correction braces (Weiss et al. 2020). Patients with scoliosis presenting to a specialist clinician before the age of six are very rare. Although there is no guideline for these patients, it is suggested that these patients are treated like those in the pubertal growth spurt, provided they are still in the growth phase (< 6 years of age).

In addition, patients benefit psychologically from intensive rehabilitation in groups during the main growth spurt period (Weiss et al. 2015). Inpatient treatment is a preferred method for patients with painful or severe pulmonary dysfunction. However, for those who have a better prognosis, out-patient sessions determined by the clinician or short-term out-patient rehabilitation prepare the patient sufficiently for home exercises. If success can be achieved by changing daily postural habits after a short rehabilitation period, these behavioural patterns may become automatic, and further intensive physical rehabilitation may not be required. It is also reported by these authors that these measures, were designed in a way that would least interfere with the quality of life of patients. (Weiss & Turnbull 2020a).

### Strengths and limitations

This is the first study to develop and validate a guideline on the conservative treatment of spinal deformities utilising the Delphi method. It includes contributions from a large number of international experts. However, the views of the Delphi panellists who agreed to participate in our study may differ from those who refused to participate and, therefore may not fully represent all expert views in the field. In addition, the decrease in the number of participants in the second Delphi round should be considered as a possible variant.

## Conclusion

Whilst the level of evidence of conservative treatment is growing, there is still a lack of a comprehensive therapeutic guideline protocol for spinal deformities’ conservative management. As tested within our study, we believe that this guideline will create a systematic method for clinicians to use in both research and clinical practice. The expert views obtained in our Delphi study showed that most of the statements were accepted in the ‘strongly agree’ level, by most of the panellists. Thus, this proposed Guideline Protocol, derived from the Schroth Best Practice Experts’ publication (Weiss & Turnbull 2020a), was approved by the Delphi method to be used as a valid tool in the management of spinal deformities.

## APPENDIX 1

### Delphi questionnaire

**Guidelines for the conservative treatment of patients with spinal deformities**

Name………………………………………..        Date…………………….

Email………………………………………

Affiliation …………………………………………………………………………………………..

……………………………………………………………………………………………………………        Country..……………..

What is your profession?………………………………..

How many years have you been working with patients with spinal deformities?

What type of patients do you see in your clinical practice?

- Scoliosis
- Adult scoliosis
- Adolescent scoliosis
- Juvenile scoliosis
- Kyphosis
- Lordosis

Which conservative treatment approaches do you use in clinical practice?

………………………………………………………………………………………………………………………………………

** Please indicate your degree of agreement or disagreement for each item (1 is little, 7 is strong Agreement/Dis agreement).*

**Table:**

Statements	Your view on (please mark)		Comments: Suggested changes, arguments, questions
	Agree	Disagree	
1. The primary goal of scoliosis and kyphosis management in growing children of Risser 0 to Risser 3 is to stop curve progression and to try to improve curvature through growth.	1 2 3 4 5 6 7	1 2 3 4 5 6 7	-
2. The primary goal of scoliosis and kyphosis management in older adolescents with less growth should be to improve cosmetic appearance and postural balance, whilst halting any further curve progression.	1 2 3 4 5 6 7	1 2 3 4 5 6 7	-
3. Improving pulmonary function (vital capacity) and treating pain are also of major importance.	1 2 3 4 5 6 7	1 2 3 4 5 6 7	-
4. Conservative scoliosis management is based on rehabilitative treatment and bracing.	1 2 3 4 5 6 7	1 2 3 4 5 6 7	-
5. Today there is evidence for the effectiveness of scoliosis treatment using physical rehabilitation alone.	1 2 3 4 5 6 7	1 2 3 4 5 6 7	-
6. Therapy for scoliosis does not just consist of general exercises.	1 2 3 4 5 6 7	1 2 3 4 5 6 7	-
7. Methods specific to scoliosis requires that clinicians be specifically trained and certified in these targeted conservative intervention methods.	1 2 3 4 5 6 7	1 2 3 4 5 6 7	-
8. Out-patient rehabilitation produces similar results to inpatient rehabilitation results and are effective at improving the common signs and symptoms of scoliosis and impeding curve progression.	1 2 3 4 5 6 7	1 2 3 4 5 6 7	-
9. Bracing is effective in preventing progression and improving curvature and in altering the natural history of idiopathic scoliosis.	1 2 3 4 5 6 7	1 2 3 4 5 6 7	-
10. Brace treatment may reduce the prevalence of surgery, restore the sagittal profile, and influence vertebral rotation.	1 2 3 4 5 6 7	1 2 3 4 5 6 7	-
11. Patient compliance is important for end-results of brace treatment.	1 2 3 4 5 6 7	1 2 3 4 5 6 7	-
12. Rigid braces have superior end-results than soft braces.	1 2 3 4 5 6 7	1 2 3 4 5 6 7	-
13. Simple deflection exercises can be performed in general to achieve a wider range of motion in kyphosis treatment.	1 2 3 4 5 6 7	1 2 3 4 5 6 7	-
14. Exercises and activities of daily living (ADLs) for patients with lumbar and thoracolumbar kyphosis are effective.	1 2 3 4 5 6 7	1 2 3 4 5 6 7	-
15. Bracing is effective in preventing curvature progression and thus in altering the natural history of kyphosis.	1 2 3 4 5 6 7	1 2 3 4 5 6 7	-
16. Each patient with scoliosis has their own natural history and must be considered on an individual basis in the context of a thorough objective clinical evaluation, patient subjective and on their past medical history.	1 2 3 4 5 6 7	1 2 3 4 5 6 7	-
17. The risk of scoliosis progression highly correlates with the potential for growth.	1 2 3 4 5 6 7	1 2 3 4 5 6 7	-
18. The progression factor should be calculated using the Lonstein and Carlson’s progression estimation formula in patients with high growth velocity.	1 2 3 4 5 6 7	1 2 3 4 5 6 7	-
19. The treatment programme should be decided by calculating the progression risk according to the age and Cobb angle in patients with lower growth velocity.	1 2 3 4 5 6 7	1 2 3 4 5 6 7	-
20. The indication for physical rehabilitation during the main growth spurt depends upon the individual and certain variables such as Cobb angle, apical curve location and Risser sign regarding the predicted treatment outcome in adolescent idiopathic scoliosis (AIS).	1 2 3 4 5 6 7	1 2 3 4 5 6 7	-
21. Brace treatment is indicated and paramount to conservative management during growth and following the main growth spurt, physical rehabilitation can be effective independently of brace treatment in AIS.	1 2 3 4 5 6 7	1 2 3 4 5 6 7	-
**For Scoliosis** ***In children (no signs of maturity, age 6–10 years)*** **Type of treatment provision:**			
22. Cobb angle up to 15° observation (6–12-month intervals).	1 2 3 4 5 6 7	1 2 3 4 5 6 7	
23. Cobb angle 15° – 20°: Physical rehabilitation with treatment-free intervals (6–12 weeks without physical rehabilitation for those patients having low risk for curve progression at the time).	1 2 3 4 5 6 7	1 2 3 4 5 6 7	
24. Cobb angle 20°–25°: Physical rehabilitation.	1 2 3 4 5 6 7	1 2 3 4 5 6 7	
25. Cobb angle > 25°: Physical rehabilitation and brace wearing part-time	1 2 3 4 5 6 7	1 2 3 4 5 6 7	
***II. In children and adolescents, Risser 0–3, first signs of maturation, less than 98 % of mature height (bone age <14 years – girls, <16 years – boys)2.*** **Type of treatment provision:**			
26. Progression risk <40%: Observation (3-month intervals).	1 2 3 4 5 6 7	1 2 3 4 5 6 7	
27. Progression risk 40% – 60%: Physical rehabilitation.	1 2 3 4 5 6 7	1 2 3 4 5 6 7	
28. Progression risk 60% – 80%: Physical rehabilitation + part-time brace indication (16 h –23 h [low risk]).	1 2 3 4 5 6 7	1 2 3 4 5 6 7	
29. Progression risk > 80%: Physical rehabilitation + full-time brace indication (22 h full time – to reduce Cobb angle and improve cosmetic appearance through growth or 16 h – 18 h part time to halt curve).	1 2 3 4 5 6 7	1 2 3 4 5 6 7	
***III. In children and adolescents presenting with Risser 4 (more than 98 % of mature height)*** **Type of treatment provision:**			
30. Cobb angle up to 20°: Observation (6–12 monthly intervals).	1 2 3 4 5 6 7	1 2 3 4 5 6 7	
31. Cobb angle 20° – 35°: Physical rehabilitation.	1 2 3 4 5 6 7	1 2 3 4 5 6 7	
32. Cobb angle > 35°: Physical rehabilitation + brace (22 h full time with aim to improve cosmetic appearance or 16 h – 18 h part time to halt curve – expectation is that the part-time wearer is not likely to improve their cobb angle at these later stages of growth).	1 2 3 4 5 6 7	1 2 3 4 5 6 7	
33. For brace weaning: Physical rehabilitation + brace with reduced wearing time.	1 2 3 4 5 6 7	1 2 3 4 5 6 7	
***IV. First presentation with Risser 4–5 (more than 99.5 % of mature height before growth is completed)*** **Type of treatment provision:**			
34. Cobb angle 25° – 35°: Physical rehabilitation.	1 2 3 4 5 6 7	1 2 3 4 5 6 7	
35. Cobb angle > 35°: Physical rehabilitation + brace (22 h full time if wanting to improve cosmetic appearance and or 16 h – 18 h part time to halt curve – expectation is that the part-time wearer is not likely to improve their cobb angle at these later stages of growth).	1 2 3 4 5 6 7	1 2 3 4 5 6 7	
*V. Adults with Cobb angles > 30°*			
36. Physical rehabilitation should be recommended.	1 2 3 4 5 6 7	1 2 3 4 5 6 7	
***VI. Adolescents and adults with scoliosis (of any degree) and chronic pain***			
37. Treatment programme should include physical rehabilitation, scoliosis rehabilitation programme (multimodal pain concept/behavioural + physical concept) and brace treatment.	1 2 3 4 5 6 7	1 2 3 4 5 6 7	
For Kyphosis			
38. Brace treatment, like in other spinal deformities, is indicated when the curvature exceeds a Cobb angle of 40° in the thoracic area and when lumbar or thoracolumbar lordosis has vanished, and / or a kyphosis is visible in these areas.	1 2 3 4 5 6 7	1 2 3 4 5 6 7	
***I. Children and adolescents, Risser 0–3, first signs of maturation, less than 98 % of mature height*** **Type of treatment provision:**			
39. If there is inhibition of extension thoracic, thoracolumbar or lumbar: Physical rehabilitation.	1 2 3 4 5 6 7	1 2 3 4 5 6 7	
40. Cobb angle > 40° thoracic, any kind of thoracolumbar or lumbar kyphosis: Physical rehabilitation + brace (Minimum brace wear of 16 h per day).	1 2 3 4 5 6 7	1 2 3 4 5 6 7	
41. When weaning from brace: Physical rehabilitation + brace with reduced wearing time.	1 2 3 4 5 6 7	1 2 3 4 5 6 7	
***II. Children and adolescents presenting with Risser 4 (more than 98 % of mature height)*** **Type of treatment provision:**	1 2 3 4 5 6 7	1 2 3 4 5 6 7	
42. Cobb angle is 40° – 50° thoracic, any kind of thoracolumbar or lumbar kyphosis: Physical rehabilitation.	1 2 3 4 5 6 7	1 2 3 4 5 6 7	
43. Cobb angle > 50° thoracic, > 10° of kyphosis thoracolumbar or lumbar: Physical rehabilitation + brace (16 h – 18 h part time if wanting to improve cosmetic appearance and halt curve – expectation is that the part-time wearer is not likely to improve their curvature at these later stages of growth).	1 2 3 4 5 6 7	1 2 3 4 5 6 7	
***III. First presentation with Risser 4–5 (more than 99.5 % of mature height before growth is completed)*** **Type of treatment provision:**			
44. Cobb angle > 50° thoracic, > 10° of kyphosis thoracolumbar or lumbar: Physical rehabilitation.	1 2 3 4 5 6 7	1 2 3 4 5 6 7	
***IV. Adults with Cobb angles thoracic > 50°, > 10° of kyphosis thoracolumbar or lumbar*** ***Type of treatment provision:***			
45. Physical rehabilitation, inpatient rehabilitation.	1 2 3 4 5 6 7	1 2 3 4 5 6 7	
***V. Adolescents and adults with kyphosis (of any degree) and chronic pain*** **Type of treatment provision:**			
46. Physical rehabilitation, scoliosis rehabilitation programme (multimodal pain concept/behavioural + physical concept), brace treatment when a positive effect has been proven during specific testing.	1 2 3 4 5 6 7	1 2 3 4 5 6 7	

*Source:* Weiss, H.R. & Turnbull, D., 2020a, ‘Best practice recommendations for the conservative treatment of patients with spinal deformities’, in M. Borysov, M. Moramarco, S.Y. Ng & Weiss, H.R. (eds.), Schroth’s textbook of scoliosis and other spinal deformities, pp. 760–775, Cambridge Scholars Publishing, Newcastle upon Tyne

## References

[CIT0001] Acaroglu, E., Guler, U.O., Cetinyurek-Yavuz, A., Yuksel, S., Yavuz, Y., Ayhan, S. et al., 2017, ‘Decision analysis to identify the ideal treatment for adult spinal deformity: What is the impact of complications on treatment outcomes?’, *Acta Orthopaedica et Traumatologica Turcica* 51(3), 181–190. 10.1016/j.aott.2017.03.003 28454778 PMC6197456

[CIT0002] Achar, S. & Yamanaka, J., 2020, ‘Back pain in children and adolescents’, *Am Fam Physician* 102(1), 19–28. 32603067

[CIT0003] Anwer, S., Alghadir, A., Abu Shaphe, M. & Anwar, D., 2015, ‘Effects of exercise on spinal deformities and quality of life in patients with adolescent idiopathic scoliosis’, *BioMed Research International* 2015, 123848. 10.1155/2015/123848 26583083 PMC4637024

[CIT0004] Berdishevsky, H., Lebel, V.A., Bettany-Saltikov, J., Rigo, M., Lebel, A., Hennes, A. et al., 2016, ‘Physiotherapy scoliosis-specific exercises – A comprehensive review of seven major schools’, *Scoliosis and Spinal Disorders* 11(1), 1–52. 10.1186/s13013-016-0076-9 27525315 PMC4973373

[CIT0005] Bettany-Saltikov, J., Turnbull, D., Ng, S.Y. & Webb, R., 2017, ‘Management of spinal deformities and evidence of treatment effectiveness’, *The Open Orthopaedics Journal* 11(Suppl 9 M6), 1521. 10.2174/1874325001711011521 29399227 PMC5759105

[CIT0006] Bettany-Saltikov, J., Weiss, H.R., Chockalingam, N., Taranu, R., Srinivas, S., Hogg, J. et al., 2015, ‘Surgical versus non-surgical interventions in people with adolescent idiopathic scoliosis’, *Cochrane Database of Systematic Reviews* (4), CD010663. 10.1002/14651858.CD010663.pub2 PMC11167694 25908428

[CIT0007] Brooks, K.W., 1979, ‘Delphi technique: Expanding applications’, *North Central Association Quarterly* 53(3), 377–385.

[CIT0008] Custer, R.L., Scarcella, J.A. & Stewart, B.R., 1999, ‘The modified Delphi technique – A rotational modification’, *Journal of Career and Technical Education* 15(2), 1–10. 10.21061/jcte.v15i2.702

[CIT0009] Dalkey, N. & Helmer, O., 1963, ‘An experimental application of the DELPHI method to the use of experts’, *Management Science* 9(3), 458–467. 10.1287/mnsc.9.3.458

[CIT0010] Day, J.M., Fletcher, J., Coghlan, M. & Ravine, T., 2019, ‘Review of scoliosis-specific exercise methods used to correct adolescent idiopathic scoliosis’, *Archives of Physiotherapy* 9(1), 8. 10.1186/s40945-019-0060-9 31463082 PMC6708126

[CIT0011] De Mauroy, J., Weiss, H., Aulisa, A., Aulisa, L., Brox, J., Durmala, J. et al., 2010, ‘7th SOSORT consensus paper: Conservative treatment of idiopathic & Scheuermann’s kyphosis’, *Scoliosis* 5(1), 9. 10.1186/1748-7161-5-9 20509962 PMC2890418

[CIT0012] Diamond, I.R., Grant, R.C., Feldman, B.M., Pencharz, P.B., Ling, S.C., Moore, A.M. et al., 2014, ‘Defining consensus: A systematic review recommends methodologic criteria for reporting of Delphi studies’, *Journal of Clinical Epidemiology* 67(4), 401–409. 10.1016/j.jclinepi.2013.12.002 24581294

[CIT0013] Diebo, B.G., Shah, N.V., Boachie-Adjei, O., Zhu, F., Rothenfluh, D.A., Paulino, C.B. et al., 2019, ‘Adult spinal deformity’, *The Lancet* 394(10193), 160–172. 10.1016/S0140-6736(19)31125-0 31305254

[CIT0014] Fernández-Llamazares, C.M., Hernández-Gago, Y., Pozas, M., Cabañas, M.J., Feal, B., Villaronga, M. et al., 2013, ‘Two-round Delphi technique for the consensual design of a paediatric pharmaceutical care model’, *Pharmacological Research: The Official Journal of the Italian Pharmacological Society* 68(1), 31–37. 10.1016/j.phrs.2012.11.001 23153856

[CIT0015] Field, M.J. & Lohr, K.N., 1992, *Guidelines for clinical practice: From development to use*, National Academies Press, Washington, DC. 25121254

[CIT0016] Goldberg, C.J., Kaliszer, M., Moore, D.P., Fogarty, E.E. & Dowling, F.E., 2001, ‘Surface topography, Cobb angles, and cosmetic change in scoliosis’, *Spine* 26, 55–63. 10.1097/00007632-200102150-00005 11224901

[CIT0017] Gür, G., Ayhan, C. & Yakut, Y., 2017, ‘The effectiveness of core stabilisation exercise in adolescent idiopathic scoliosis: A randomised controlled trial’, *Prosthetics and Orthotics International* 41(3), 303–310. 10.1177/0309364616664151 27625122

[CIT0018] Herman, R., Mixon, J., Fisher, A., Maulucci, R. & Stuyck, J., 1985, ‘Idiopathic scoliosis and the central nervous system: A motor control problem: The Harrington lecture, 1983 scoliosis research society’, *Spine* 10(1), 1–14. 10.1097/00007632-198501000-00001 3885413

[CIT0019] Johari, J., Sharifudin, M.A., Ab Rahman, A., Omar, A.S., Abdullah, A.T., Nor, S. et al., 2016, ‘Relationship between pulmonary function and degree of spinal deformity, location of apical vertebrae and age among adolescent idiopathic scoliosis patients’, *Singapore Medical Journal* 57(1), 33–38. 10.11622/smedj.2016009 26831315 PMC4728701

[CIT0020] Kaspiris, A., Grivas, T.B., Weiss, H.R. & Turnbull, D., 2011, ‘Surgical and conservative treatment of patients with congenital scoliosis: α search for long-term results’, *Scoliosis* 6(1), 1–17. 10.1186/1748-7161-6-12 21639924 PMC3120793

[CIT0021] Kim, H.J., Yang, J.H., Chang, D.G., Suk, S.I., Suh, S.W., Song, K.S. et al., 2020, ‘Adult spinal deformity: Current concepts and decision-making strategies for management’, *Asian Spine Journal* 14(6), 886–897. 10.31616/asj.2020.0568 33254357 PMC7788366

[CIT0022] Kostuik, J.P., 2015, ‘The history of spinal deformity’, *Spine Deformity* 3(5), 417–425. 10.1016/j.jspd.2015.07.003 27927528

[CIT0023] Kuru, T. & Yilmaz, H., 2012, ‘Assessment of stress in adolescent idiopathic scoliosis patients while wearing a brace’, *Scoliosis* 7(S1), O4. 10.1186/1748-7161-7-S1-O4

[CIT0024] Li, X., Shen, J., Liang, J., Zhou, X., Yang, Y., Wang, D. et al., 2021, ‘Effect of core-based exercise in people with scoliosis: A systematic review and meta-analysis’, *Clinical Rehabilitation* 35(5), 669–680. 10.1177/0269215520975105 33356498 PMC8076838

[CIT0025] Liu, D., Yang, Y., Yu, X., Yang, J., Xuan, X., Yang, J. et al., 2020, ‘Effects of specific exercise therapy on adolescent patients with idiopathic scoliosis: A prospective controlled cohort study’, *Spine* 45(15), 1039–1046. 10.1097/BRS.0000000000003451 32675606 PMC7373466

[CIT0026] Loughlin, K.G. & Moore, L.F., 1979, ‘Using Delphi to achieve congruent objectives and activities in a pediatrics department’, *Journal of Medical Education* 54(2), 101–106. 10.1097/00001888-197902000-00006 762686

[CIT0027] Marks, D.S. & Qaimkhani, S.A., 2009, ‘The natural history of congenital scoliosis and kyphosis’, *Spine* 34(17), 1751–1755. 10.1097/BRS.0b013e3181af1caf 19644326

[CIT0028] Menendez, J.Y., Omar, N.B., Chagoya, G., Tabibian, B.E., Elsayed, G.A., Walters, B.C. et al., 2019, ‘Patient satisfaction in spine surgery: A systematic review of the literature’, *Asian Spine Journal* 13(6), 1047–1057. 10.31616/asj.2019.0032 31352720 PMC6894977

[CIT0029] Meshkat, B., Cowman, S., Gethin, G., Ryan, K., Wiley, M., Brick, A. et al., 2014, ‘Using an e-Delphi technique in achieving consensus across disciplines for developing best practice in day surgery in Ireland’, *Journal of Hospital Administration* 3(4), 1–8. 10.5430/jha.v3n4p1

[CIT0030] Minsk, M.K., Venuti, K.D., Daumit, G.L. & Sponseller, P.D., 2017, ‘Effectiveness of the Rigo Chêneau versus Boston-style orthoses for adolescent idiopathic scoliosis: A retrospective study’, *Scoliosis and Spinal Disorders* 12(1), 7. 10.1186/s13013-017-0117-z 28331904 PMC5357818

[CIT0031] Murphy, M.K., Black, N.A., Lamping, D.L., McKee, C.M., Sanderson, C.F.B, Askham, J. et al., 1998, ‘Consensus development methods, and their use in clinical guideline development’, *Health Technology Assessment* 2(3), 87–88. 10.1177/135581969900400410 9561895

[CIT0032] Negrini, A., Negrini, M.G., Donzelli, S., Romano, M., Zaina, F. & Negrini, S., 2015, ‘Scoliosis-specific exercises can reduce the progression of severe curves in adult idiopathic scoliosis: A long-term cohort study’, *Scoliosis* 10(1), 1–7. 10.1186/s13013-015-0044-9 26279670 PMC4537533

[CIT0033] Negrini, S., Antonini, G., Carabalona, R. & Minozzi, S., 2003, ‘Physical exercises as a treatment for adolescent idiopathic scoliosis: A systematic review’, *Pediatric Rehabilitation* 6(3–4), 227–235. 10.1080/13638490310001636781 14713590

[CIT0034] Negrini, S., Donzelli, S., Aulisa, A.G., Czaprowski, D., Schreiber, S., De Mauroy, J.C. et al., 2018, ‘2016 SOSORT guidelines: Orthopaedic and rehabilitation treatment of idiopathic scoliosis during growth’, *Scoliosis and Spinal Disorders* 13, 3. 10.1186/s13013-017-0145-8 29435499 PMC5795289

[CIT0035] Park, J.H., Jeon, H.S. & Park, H.W., 2017, ‘Effects of the Schroth exercise on idiopathic scoliosis: A meta-analysis’, *European Journal of Physical and Rehabilitation Medicine* 54(3), 440–449. 10.23736/S1973-9087.17.04461-6 28976171

[CIT0036] Parveen, A., Nuhmani, S., Ejaz Hussain, M. & Hussain Khan, M., 2020, ‘Effect of lumbar stabilisation exercises and thoracic mobilisation with strengthening exercises on pain level, thoracic kyphosis, and functional disability in chronic low back pain’, *Journal of Complementary & Integrative Medicine*. 10.1515/jcim-2019-0327 32712591

[CIT0037] Patton, M.Q., 2008, *Utilization-focused evaluation*, 4th edn., Sage Publications, Thousand Oaks, CA.

[CIT0038] RAND Corporation, 2021, *History and Mission*, viewed 27 November 2021, from https://www.rand.org/about/history.html

[CIT0039] Rigo, M., Reiter, C. & Weiss, H.R., 2003, ‘Effect of conservative management on the prevalence of surgery in patients with adolescent idiopathic scoliosis’, *Pediatric Rehabilitation* 6(3–4), 209–214. 10.1080/13638490310001642054 14713587

[CIT0040] Rivett, L., Stewart, A. & Potterton, J., 2014, ‘The effect of compliance to a Rigo System Cheneau brace and a specific exercise programme on idiopathic scoliosis curvature: A comparative study: SOSORT 2014 award winner’, *Scoliosis* 9, 5. 10.1186/1748-7161-9-5 24926318 PMC4055379

[CIT0041] Romano, M., Minozzi, S., Zaina, F., Saltikov, J.B., Chockalingam, N., Kotwicki, T. et al., 2013, ‘Exercises for adolescent idiopathic scoliosis: A cochrane systematic review’, *Spine* 38(14), E883–E893. 10.1097/BRS.0b013e31829459f8 23558442

[CIT0042] Schultz, A.B., 1984, ‘Biomechanical factors in the progression of idiopathic scoliosis’, *Annals of Biomedical Engineering* 12(6), 621–630. 10.1007/BF02371453 6398636

[CIT0043] Stewart, D., Gibson-Smith, K., MacLure, K., Mair, A., Alonso, A., Codina, C. et al., 2017, ‘A modified Delphi study to determine the level of consensus across the European Union on the structures, processes and desired outcomes of the management of polypharmacy in older people’, *PLoS One* 12(11), e0188348. 10.1371/journal.pone.0188348 29155870 PMC5695766

[CIT0044] Veraar, C., Hasler, P. & Schirmer, M., 2018, ‘A multidisciplinary Delphi consensus-based checklist to define clinical documentation tools for both routine and research purposes’, *Health Services Research and Managerial Epidemiology* 5, 1–5. 10.1177/2333392817754161 PMC5808956 29450216

[CIT0045] Weinstein, S.L., Dolan, L.A., Wright, J.G. & Dobbs, M.B., 2013, ‘Effects of bracing in adolescents with idiopathic scoliosis’, *The New England Journal of Medicine* 369(16), 1512–1521. 10.1056/NEJMoa1307337 24047455 PMC3913566

[CIT0046] Weiss, H.R., 2010, ‘Spinal deformities rehabilitation – State of the art review’, *Scoliosis* 5, 28. 10.1186/1748-7161-5-28 21184673 PMC3023759

[CIT0047] Weiss, H.R., 2011, ‘The method of Katharina Schroth – History, principles and current development’, *Scoliosis* 6(1), 17. 10.1186/1748-7161-6-17 21878114 PMC3180431

[CIT0048] Weiss, H.R., Lay, M., Seibel, S. & Kleban, A., 2020, ‘Ist eine Verbesserung der Behandlungssicherheit in der Korsettversorgung von Skoliosepatienten durch Anwendung standardisierter CAD-Algorithmen möglich? [Is it possible to improve treatment safety in the brace treatment of scoliosis patients by using standardized CAD algorithms?]’, *Der Orthopade* 50, 435–445. 10.1007/s00132-020-04000-9 PMC8189986 33025038

[CIT0049] Weiss, H.R., Lehnert-Schroth, C., Moramarco, M. & Moramarco, K., 2015, *Schroth therapy: Advancements in conservative Scoliosis treatment*, Lap Lambert Academic Publishing, Atlanta, GA.

[CIT0050] Weiss, H.R. & Moramarco, M., 2013, ‘Indication for surgical treatment in patients with adolescent Idiopathic Scoliosis – A critical appraisal’, *Patient Safety in Surgery* 7(1), 17. 10.1186/1754-9493-7-17 23705983 PMC3668989

[CIT0051] Weiss, H.R. & Moramarco, M., 2017, ‘The changing paradigm in the management of spinal deformities’, *The Open Orthopaedics Journal* 11, 1449–1451. 10.2174/1874325001711011449 29399222 PMC5759106

[CIT0052] Weiss, H.R., Negrini, S., Rigo, M., Kotwicki, T., Hawes, M.C., Grivas, T.B., et al., 2006, ‘Indications for conservative management of scoliosis (guidelines)’, *Scoliosis* 1(1), 5. 10.1186/1748-7161-1-5 16759357 PMC1479370

[CIT0053] Weiss, H.R. & Turnbull, D., 2019, ‘Non-specific chronic low back pain in patients with scoliosis – An overview of the literature on patients undergoing brace treatment’, *Journal of Physical Therapy Science* 31(11), 960–964. 10.1589/jpts.31.960 31871385 PMC6879412

[CIT0054] Weiss, H.R. & Turnbull, D., 2020a, ‘Best practice recommendations for the conservative treatment of patients with spinal deformities’, in M. Borysov, M. Moramarco, S.Y. Ng & Weiss, H.R. (eds.), *Schroth’s textbook of scoliosis and other spinal deformities*, pp. 760–775, Cambridge Scholars Publishing, Newcastle upon Tyne.

[CIT0055] Weiss, H.R. & Turnbull, D., 2020b, ‘Brace treatment for children and adolescents with scoliosis’, in *Spinal deformities in adolescents, adults and older adults*, IntechOpen, viewed from https://www.intechopen.com/online-first/brace-treatment-for-children-and-adolescents-with-scoliosis?fbclid=IwAR223NHkjaah5vYra6mK70x-kvAk7P1M_zA-iE4uWwirETgP3vglXqJc1EM.

[CIT0056] Weiss, H.R., Turnbull, D., Tournavitis, N. & Borysov, M., 2016, ‘Treatment of scoliosis-evidence and management (review of the literature)’, *Middle East Journal of Rehabilitation and Health* 3(2), e35377. 10.17795/mejrh-35377

[CIT0057] Weiss, H.R., Weiss, G. & Petermann, F., 2003, ‘Incidence of curvature progression in idiopathic scoliosis patients treated with scoliosis in-patient rehabilitation (SIR): An age- and sex-matched controlled study’, *Pediatric Rehabilitation* 6(1), 23–30. 10.1080/1363849031000095288 12745892

[CIT0058] Williams, P.L. & Webb, C., 1994, ‘Clinical supervision skills: A Delphi and critical incident technique study’, *Medical Teacher* 16(2–3), 139–157. 10.3109/01421599409006725

[CIT0059] Woolf, S.H., Grol, R., Hutchinson, A., Eccles, M. & Grimshaw, J., 1999, ‘Clinical guidelines: Potential benefits, limitations, and harms of clinical guidelines’, *BMJ* 318(7182), 527–530. 10.1136/bmj.318.7182.527 10024268 PMC1114973

